# Effect of Dynamic Recrystallization on Microstructural Evolution in B Steels Microalloyed with Nb and/or Mo

**DOI:** 10.3390/ma15041424

**Published:** 2022-02-15

**Authors:** Irati Zurutuza, Nerea Isasti, Eric Detemple, Volker Schwinn, Hardy Mohrbacher, Pello Uranga

**Affiliations:** 1 Materials and Manufacturing Division, CEIT-Basque Research and Technology Alliance (BRTA), 20018 Donostia-Saint Sebastian, Basque Country, Spain; izurutuza@ceit.es (I.Z.); nisasti@ceit.es (N.I.); 2 Mechanical and Materials Engineering Department, Universidad de Navarra, Tecnun, 20018 Donostia-Saint Sebastian, Basque Country, Spain; 3 Aktien-Gesellschaft der Dillinger Hüttenwerke, 66763 Dillingen/Saar, Germany; Eric.Detemple@dillinger.biz (E.D.); Volker.Schwinn@dillinger.biz (V.S.); 4 NiobelCon BV, 2970 Schilde, Belgium; hm@niobelcon.net; 5 Department of Materials Engineering (MTM), KU Leuven, 3001 Leuven, Belgium

**Keywords:** austenite conditioning, multipass torsion tests, dynamic recrystallization, Nb–Mo-microalloyed steels

## Abstract

The dynamic recrystallization behavior of ultra-high strength boron-microalloyed steels optionally alloyed with niobium and molybdenum is analyzed in this paper. Multipass torsion tests were performed to simulate plate rolling conditions followed by direct quenching. The influence of alloy composition on the transformed microstructure was evaluated by means of EBSD, thereby characterizing the morphology of the austenite grain morphology after roughing and finishing passes. The results indicated that for Nb-microalloyed steel, partial dynamic recrystallization occurred and resulted in local clusters of fine-sized equiaxed grains dispersed within the pancaked austenitic structure. A recrystallized austenite fraction appeared and transformed into softer phase constituents after direct quenching. The addition of Mo was shown to be an effective means of suppressing dynamic recrystallization. This effect of molybdenum in addition to its established hardenability effects hence safeguards the formation of fully martensitic microstructures, particularly in direct quenching processes. Additionally, the circumstances initiating dynamic recrystallization were studied in more detail, and the interference of the various alloying elements with the observed phenomena and the potential consequences of dynamic recrystallization before quenching are discussed.

## 1. Introduction

Ultra-high strength steel with a martensitic microstructure is the preferred material for structural applications requiring an extreme load-bearing capacity or superior wear resistance. Martensitic steels are traditionally produced by conventional quenching (CQ), where the steel is reheated from ambient temperature back into austenite before quenching. Direct quenching (DQ) is an increasingly often practiced variant for processing ultra-high strength steel that enables cost and capacity optimization in steel mills [1]. The DQ method typically applies fast cooling to conditioned austenite, while the CQ method acts on a normalized (equiaxed) austenite microstructure. Accordingly, the martensite substructure originating from the DQ process develops within a pancaked austenite microstructure [2].

The microstructural homogeneity of austenite before quenching is related to recrystallization phenomena occurring along the entire austenite hot working process. An inhomogeneous prior austenite microstructure is detrimental for the toughness and (particularly) ductile-to-brittle transition temperature of the quenched steel [2,3]. Microstructural heterogeneity in austenite can be generated at different stages during the hot working process. Alloy additions of boron, niobium, and molybdenum induce strong solute drag on austenite boundaries, thus delaying austenite recrystallization at temperatures between 1000 and 1100 °C [4]. If the austenite temperature during the last passes of recrystallizing rolling (roughing) drops into that range, the recrystallization of austenite, especially in the plate center, may not completely occur and individual non-recrystallized grains may not be as refined as the recrystallized ones. This heterogeneity cannot be removed by subsequent austenite conditioning (pancaking), resulting in pancaked grains of different thicknesses. Only a complete normalization, as occurs under CQ conditions, results in a homogeneous austenite microstructure.

On the other hand, strong austenite conditioning, which is typically connoted with high reduction ratio and low finishing temperatures, can trigger dynamic recrystallization in part of a microstructure. This event produces a fraction of very fine equiaxed austenite grains. It has been shown that the application of large deformation strain at low austenite temperature and the presence of dynamically recrystallized austenite compromises the hardenability effect related to boron microalloying [2,5,6,7,8]. The hardenability related to molybdenum alloying, however, appears to be much more robust under the same processing conditions. Although the impacts of Nb and Mo in dynamic recrystallization kinetics have been already analyzed, the synergetic effect of Nb, Mo and B for higher Mo contents needs to be further explored. Therefore, the authors of the current study investigates the circumstances initiating dynamic recrystallization in more detail. The interference of the various alloying elements (Mo, Nb, and B) with the observed phenomena and potential consequences of dynamic recrystallization before quenching are discussed.

## 2. Materials and Methods

The chemical composition of the boron-microalloyed steel designs using individual or combined Nb and Mo additions is listed in Table 1. Boron was stopped from forming boron nitride with an appropriate microalloy addition of titanium. The CMnB steel was used as a reference and for comparison based on previous papers.

**Figure 1:**
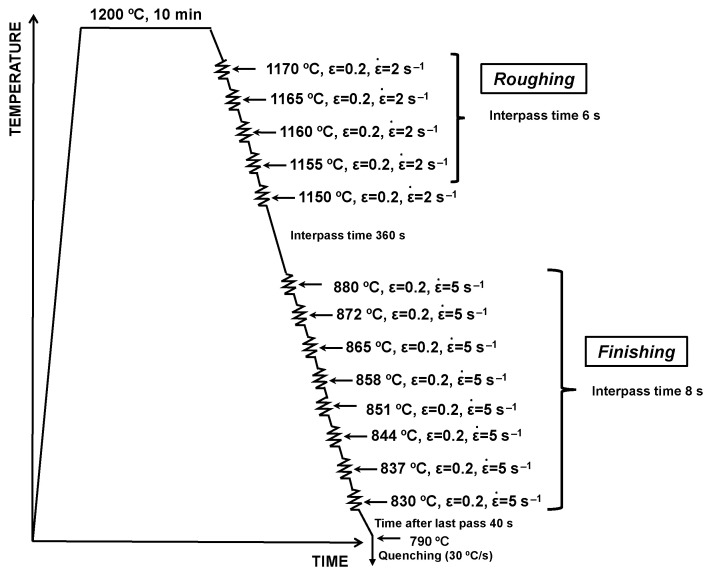
Schematics of multipass thermomechanical cycle employed with the torsion testing machine for simulating plate hot rolling.

Multipass torsion tests were performed to carry out hot rolling simulations followed by direct quenching. The torsion samples comprised a reduced central gauge section of 17 mm in length with a diameter of 7.5 mm. The torsion specimens were subjected to the thermomechanical deformation schedule shown in Figure 1. After soaking at 1200 °C for 10 min, allowing for the nearly complete dissolution of the microalloying elements B and Nb, five deformation passes with the austenite temperature gradually decreasing from 1170 to 1150 °C were executed in order to re-produce the roughing stage. In the roughing passes, a deformation strain of 0.2 at a strain rate of 2 s^−1^ and an interpass time of 6 s was applied. Between roughing and finishing, the material was held for 360 s, allowing for cooling to a finishing–start temperature of 880 °C. Eight finish deformation passes, each applying a strain of 0.2, were applied with a strain rate of 5 s^−1^. The finish deformation sequence ended at 830 °C. Subsequent slow cooling (1 °C/s) until 790 °C was followed by accelerated cooling at a rate of approximately 30 °C/s down to ambient temperature.

**Figure 2:**
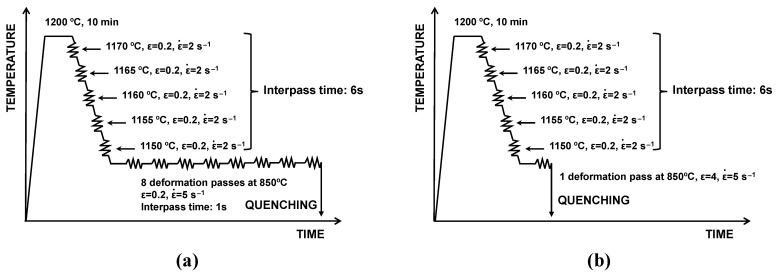
Thermomechanical schedules employed at the torsion testing machine for analyzing dynamic recrystallization phenomena: (**a**) roughing simulation followed by 8 deformation passes at 850 °C (*ε* = 0.2 and $\dot{\varepsilon }\;$ = 5 s^−1^) and (**b**) roughing simulation followed by a deformation pass at 850 °C (*ε* = 4 and $\dot{\varepsilon \;}$ = 5 s^−1^).

Two specific hot torsion schedules (Figure 2) were designed to evaluate the influence of the alloying elements Nb and Mo on the occurrence of dynamic recrystallization and to verify under which conditions this mechanism was triggered during finishing deformation passes. Both thermomechanical cycles started from reheating at 1200 °C for 10 min, followed by five roughing passes, similar to the ones defined for the hot rolling simulation (Figure 1). In one schedule (Figure 2a), the samples were cooled down to 850 °C after the last roughing pass, when 8 finish deformation passes were isothermally applied with a strain of 0.2, a strain rate of 5 s^−1^ and an interpass time of 1 s. The other schedule (Figure 2b) consisted of one large deformation cycle at 850 °C, with a strain of 4 and a strain rate of 5 s^−1^. Both schedules were followed by quenching to room temperature with a rate of approximately 30 °C/s.

The quenched martensitic microstructures were metallographically characterized in the sub-surface longitudinal section, corresponding to 0.9 of the outer radius of the torsion specimen. The analysis of the austenite structure was performed after etching in 2% Nital by optical microscopy (OM, LEICA DM1500 M, Leica microsystems, Wetzlar, Germany), and the quantification of microstructural features was performed via electron backscattered diffraction (EBSD). The EBSD samples were polished down to 1 µm, and the final polishing was performed with colloidal silica. EBSD was performed on the equipment with a camera NORDLYS II (Oxford Instruments, Abingdon, UK), a well as an acquisition program and data analysis, OXFORD HKL CHANNEL 5 PREMIUM coupled to the JEOL JSM-7100 F FEG-SEM (JEOL Ltd., Tokyo, Japan). A scan step size of 0.2 µm was used, and a total scanned area of 140 × 140 µm^2^ was defined for characterization of martensitic microstructure. The EBSD scans were analyzed by means of TSL OIM™ Analysis 5.31 software (EDAX, Mahwah, NJ, USA).

Besides analyzing the direct quenched martensite, the austenitic structures prior to martensite transformation were also characterized after etching in a solution of saturated picric acid HCl. Due to the highly deformed austenitic microstructure, the reconstruction of austenite was carried out by means of EBSD. For reconstructing the austenite prior to transformation, a scan step size of 1 µm and a total scanned area of 500 × 500 µm^2^ were defined. The analysis of strain-induced precipitation was also performed in the sub-surface longitudinal section of the torsion specimen using a transmission electron microscope (TEM, JEOL 2100, JEOL Ltd., Tokyo, Japan) with a voltage of 200 kV and LaB6 thermionic filament. This analysis was done using carbon extraction replicas.

## 3. Results

### 3.1. Characterization of the Direct Quenched Martensitic Microstructure after Plate Hot Rolling Simulation

**Figure 3:**
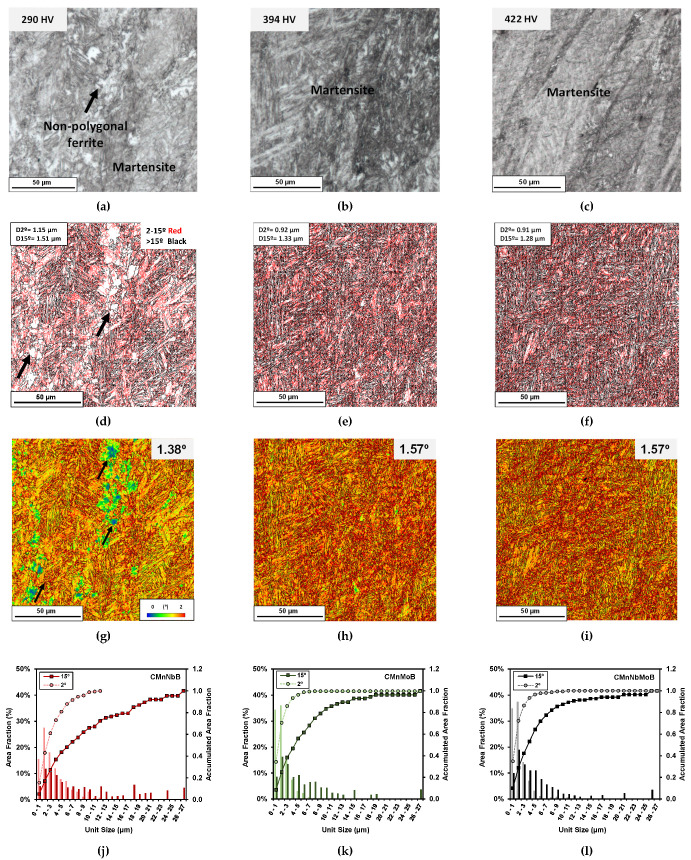
(**a**–**c**) Optical images, (**d**–**f**) grain boundary maps, and (**g**–**i**) kernel maps obtained for (**a**,**d**,**g**) CMnNbB, (**b**,**e**,**h**) CMnMoB, and (**c**,**f**,**i**) CMnNbMoB steels. (**j**–**l**) Unit size distributions measured for Nb, Mo, and NbMo grades, respectively (low and high angle misorientation criteria are considered).

The microstructures of the direct quenched steels following the hot deformation illustrated in Figure 1 are shown in Figure 3a–c. While the Mo and NbMo added steels exhibited fully martensitic microstructures, the CMnNbB steel comprised clusters consisting of non-polygonal ferrite within the martensitic microstructure. The presence of the ferrite phase resulted in a rather low hardness of 290 HV. The Mo- and NbMo-alloyed steels had much higher hardness values of 394 and 422 HV, respectively.

EBSD analysis was performed in the same specific locations corresponding to the optical images. Figure 3d–f shows grain boundary maps, in which low angle (between 2° and 15°) and high angle boundaries (>15°) are represented by red and black colors, respectively. Grain boundary maps corresponding to the three steel grades confirm the formation of a very fine-sized and complex microstructure. The presence of a substructure is reflected in a high density of low angle boundaries. However, in the CMnNbB steel (Figure 3d), some areas are clearly lacking this substructure, thus indicating that a softer phase was formed within the otherwise martensitic matrix. The mean unit sizes considering both tolerance angles (D2° and D15°) are indicated in the grain boundary maps. The CMnMoB and CMnNbMoB steels showed similar mean unit sizes, D2° and D15°, of about 0.9 and 1.3 μm, respectively. However, for CMnNbB grades, slightly coarser mean unit sizes of 1.1 and 1.5 μm were found for D2° and D15°, respectively. The kernel average misorientation (KAM) maps shown in Figure 3g–i reflect the presence of highly dislocated microstructures such as martensite and bainite (red- and yellow-colored areas, respectively). Yet in the CMnNbB steel, larger islands with lower dislocation density (blue and green colored areas) can be seen; these represent softer ferritic phases. The KAM value increased from 1.38° in CMnNb steel to 1.57° in the CMnMoB and CMnNbMo steels. In Figure 3j–l, the unit size distributions considering low and high angle misorientation criteria (boundaries between 2° and 15° and boundaries higher than 15°, respectively) are plotted for the three steels. For both misorientation criteria, a finer unit size distribution can be observed in the CMnNbMoB steel compared to the CMnNbB steel.

### 3.2. Impact of Adding Nb and Mo on Austenite Conditioning

**Figure 4:**
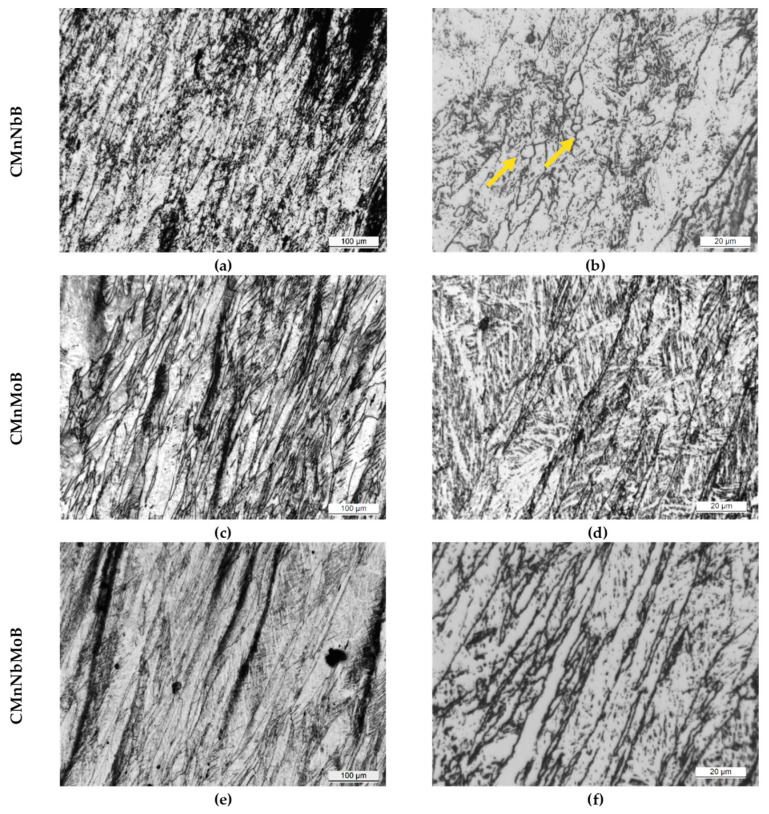
Optical micrographs at different magnifications ((**a**,**c**,**e**) and (**b**,**d**,**f)**, at low and high magnifications, respectively) corresponding to all steel grades after the multipass torsion test (etched by Picric acid).

As the final martensitic features are strongly influenced by the austenite morphology prior to phase transformation, the characterization of the prior austenitic structure was analyzed (Figure 4). However, hot rolling simulations generating extremely deformed austenite hinder quantitative characterization. In all steel alloys, the microstructure comprised highly elongated austenite grains (Figure 4a,c,e). The accumulation of deformation was most pronounced for the combined addition of Nb and Mo (Figure 4e,f). Analyzing the optical images obtained at higher magnifications (Figure 4b,d,f) revealed a fraction of fine equiaxed grains in the CMnNbB grade, as indicated with red arrows in Figure 4b. These must have resulted from localized dynamic recrystallization occurring during final deformation. In the Mo-bearing steel grades, such equiaxed grains were not observed (see Figure 4d,f).

**Figure 5:**
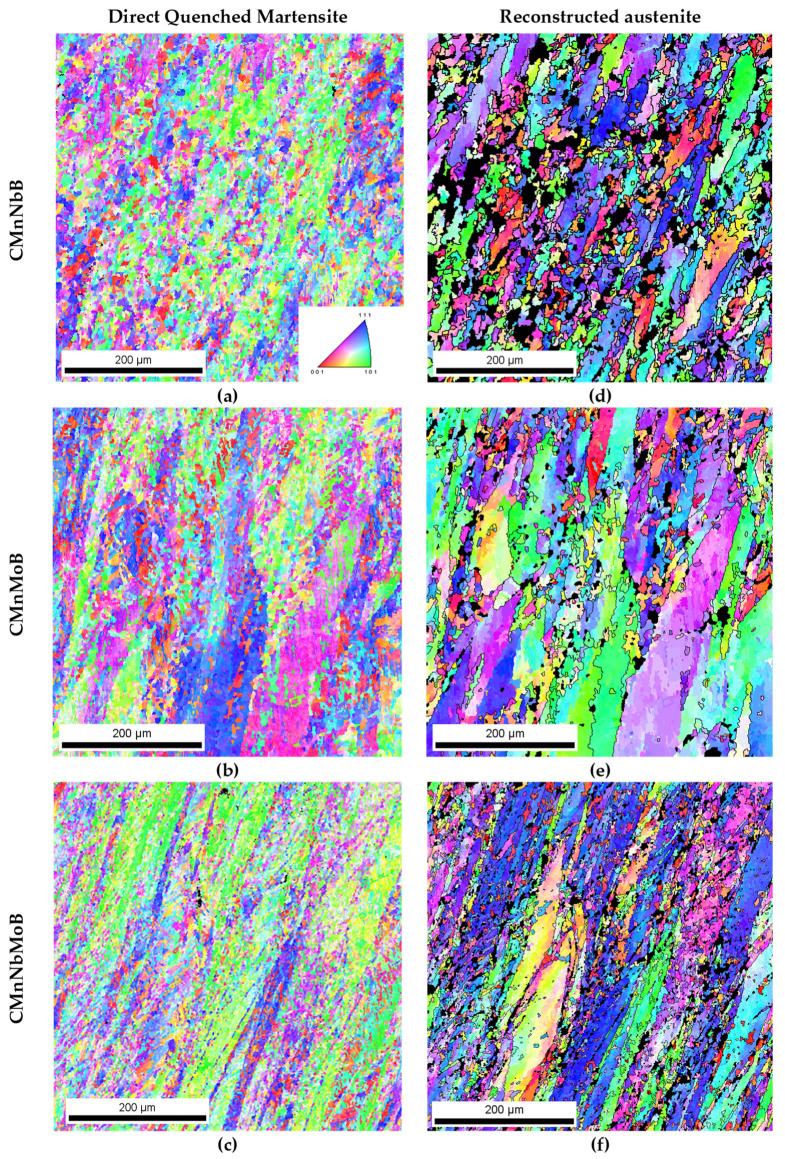
(**a**–**c**) IPF maps corresponding to the martensitic microstructure and (**d**–**f**) Reconstructed austenite microstructures for different steel grades.

EBSD inverse pole figure (IPF) maps obtained on the martensitic microstructures (Figure 5a–c) allowed for the reconstruction of the prior austenite grain structure (Figure 5d–f) according to a procedure defined in [9,10]. The reconstruction confirmed the presence of elongated austenite grains in all steels. In agreement with the optical microscopy analysis, the reconstructed austenite structure of the CMnNbB steel demonstrated a fraction of very fine equiaxed grains.

**Figure 6:**
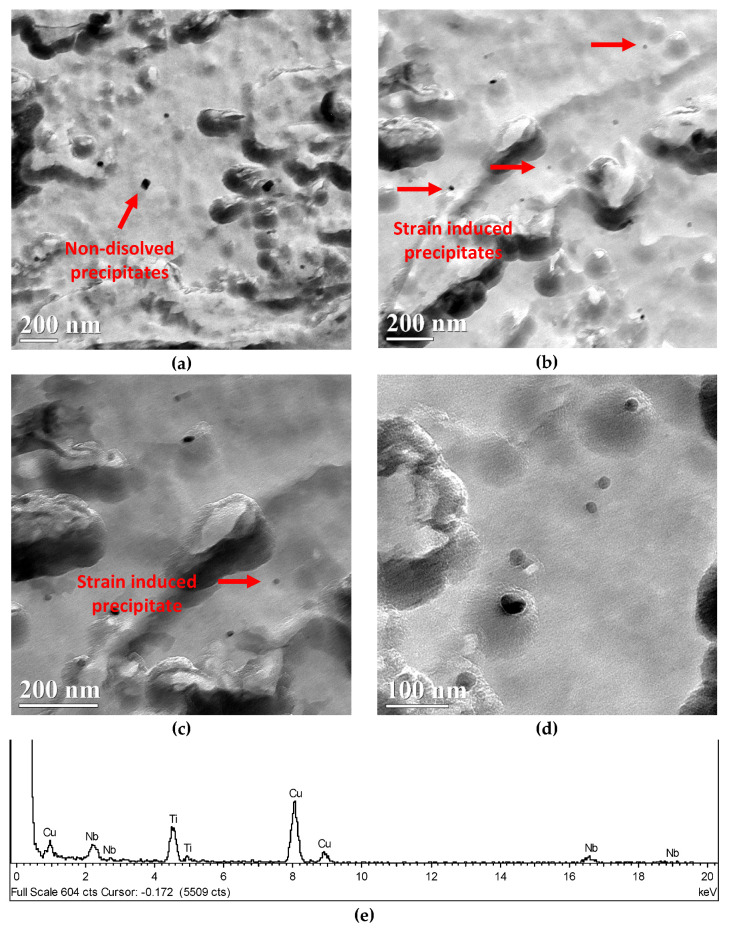
(**a**–**d**) Presence of non-dissolved Nb–Ti precipitates and strain-induced fine precipitates (NbTi-rich) in CMnNbB steel after plate hot rolling simulation and DQ. (**e**) Microanalysis of the strain-induced precipitate marked in (**c**) with a red arrow (the presence of Cu in the spectrum is associated with the grid holding of the carbon replica).

When deforming Nb-microalloyed steels at austenite temperatures below T_nr_, strain-induced precipitates can be formed, delaying static recrystallization and promoting strain accumulation prior to transformation. Usually, the pinning effect of these strain-induced particles is assumed to be strong enough to block any further static recrystallization during conventional hot deformation sequences. Carbon extraction replicas of the CMnNbB steel were analyzed with TEM, as shown in Figure 6, where different precipitate populations can be identified. Precipitates composed of Nb and Ti of relatively larger size (Figure 6a) were likely undissolved particles existing prior to the soaking treatment. Additionally, strain-induced precipitates in the size range between 10 and 30 nm were observed (Figure 6b–d). The microanalysis shown in Figure 6e shows that Nb and Ti comprised these carbide particles. These strain-induced precipitates efficiently blocked static recrystallization during the finishing passes.

Molybdenum, on the other hand, does not precipitate in austenite due to its high solubility even for the current addition of 0.5 mass% [11]. Hence, the observed austenite pancaking in the CMnMoB steel must have primarily been caused by a strong solute drag effect acting on the grain boundaries. The absence of fine-sized equiaxed austenite grains in the microstructure of that steel suggests that the presence of strain-induced precipitates is not decisive for the avoidance of dynamic recrystallization.

### 3.3. Analysis of Dynamic Recrystallization Onset

**Figure 7:**
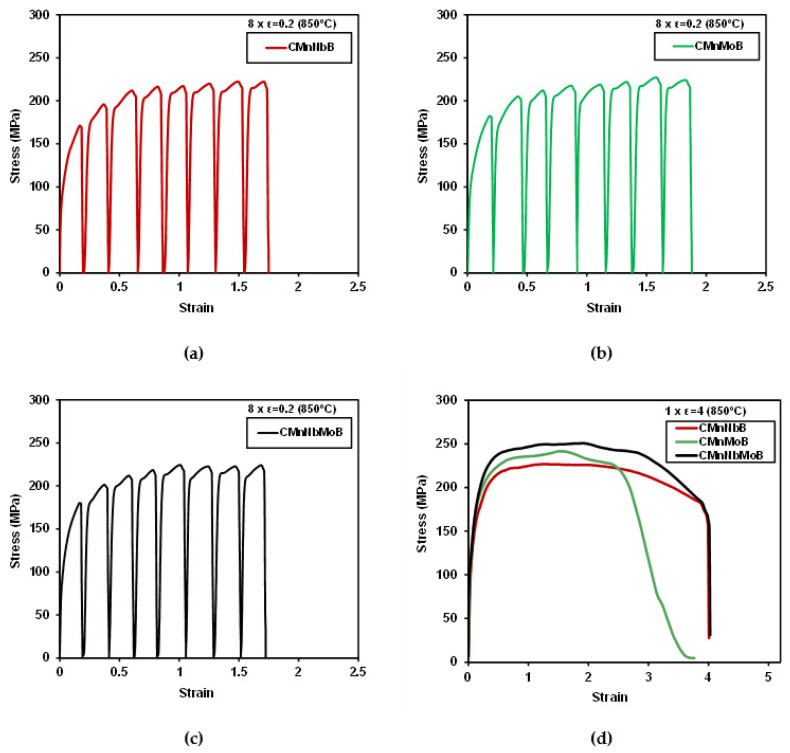
(**a**–**c**) Stress–strain curves for 8 deformation passes of *ε* = 0.2 (CMnNbB, CMnMoB, and CMnNbMoB grades, respectively) and (**d**) one deformation pass of *ε* = 4 at 850 °C.

**Figure 8:**
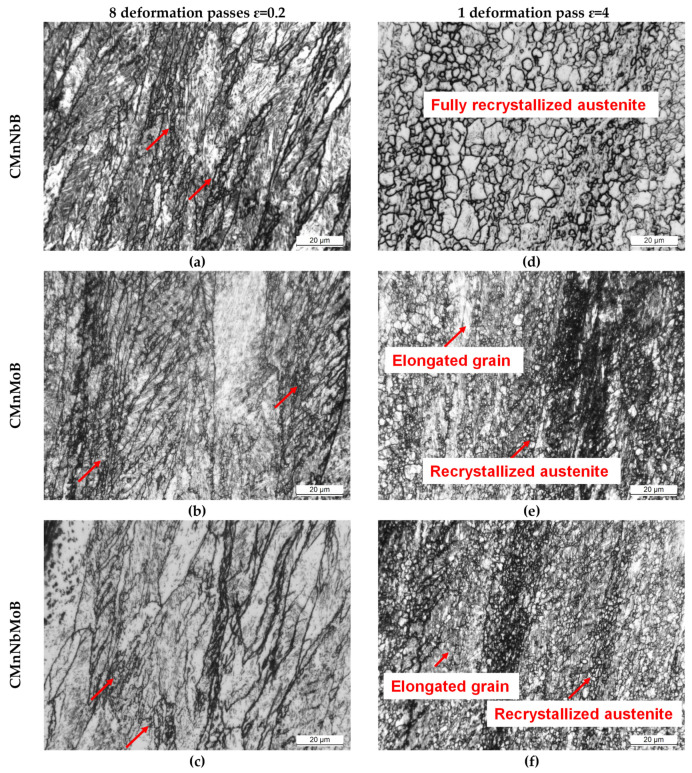
Optical images corresponding to the austenitic structure obtained after the thermomechanical cycle shown in Figure 2: (**a**–**c**) Roughing simulation and 8 deformation passes of 0.2 at 850 °C (see Figure 2a); (**d**–**f**) roughing simulation and 1 deformation pass of 4 at 850 °C (see Figure 2b). Dynamically recrystallized grains are indicated with red arrows.

The deformation schedule specified in Figure 2a was designed to provoke dynamic recrystallization in the investigated steels. The resulting stress–strain curves in Figure 7a–c indicate that the peak stress was reached during the fifth deformation pass for the Nb-microalloyed steels and during the seventh deformation pass for the CMnMoB steel. The molybdenum-alloyed steels reached a higher peak stress than the CMnNbB steel. The stress–strain curve reveals a transition from continuous yielding to pronounced yielding in the second pass for the Nb-microalloyed steels. This could have been related to the strain-induced precipitation of Nb. The Nb-free steel only showed this yielding phenomenon during later passes. Potentially, Ti or B formed precipitates in that steel since molybdenum does not form carbides in austenite due to its good solubility. Analyzing the austenite grain structure after eight deformation passes (Figure 8a–c) revealed a fraction of extremely fine-sized equiaxed austenite grains within the pancaked austenite matrix of all steels, as indicated with red arrows in Figure 8a. The recrystallized austenite grains were clustered in areas where austenite pancakes were particularly thin. Apparently, molybdenum alloying could not completely prevent the initiation of dynamic recrystallization. However, molybdenum significantly suppressed the volume fraction of recrystallized austenite grains during the simulated plate rolling schedule (Figure 5).

**Figure 9:**
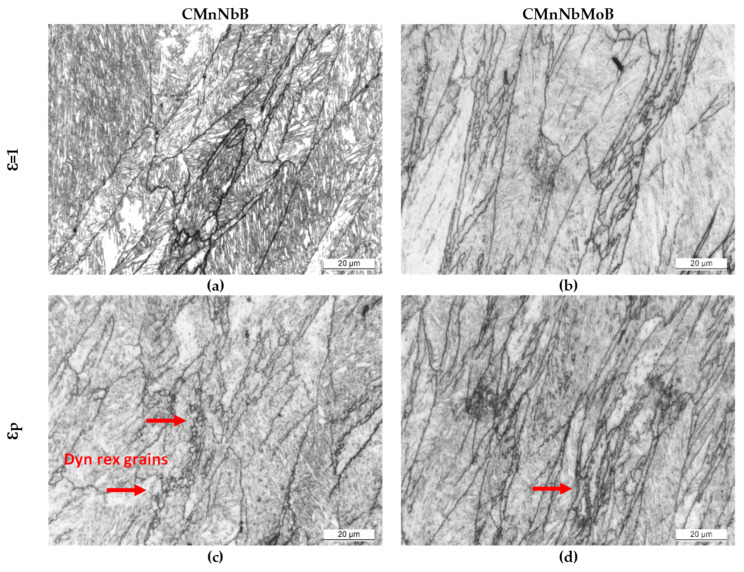
Optical micrographs after (**a**,**b**) *ε* = 1 and (**c**,**d**) *ε*_p_ deformation passes for: (**a**,**c**) CMnNbB and (**b**,**d**) CMnNbMoB steel grades (dynamically recrystallized grains are indicated with red arrows).

This effect was further elucidated after applying a large strain during a single deformation pass according to Figure 2b. The stress–strain curves of all three steels reveal continuous yielding (Figure 7d). The mean flow stress increased in the order of CMnNbB, CMnMoB, and CMnMoNbB steels. The austenite grain structure after the large deformation cycle (Figure 8d–f) reflected the nearly complete recrystallization in the CMnNbB steel, which showed fine-sized equiaxed grains with sizes of up to around 10 µm. The molybdenum-alloyed steels, however, presented a mixed microstructure consisting of elongated grains and recrystallized grains. The recrystallized grains were extremely fine-sized, typically smaller than 3 µm. This observation again indicates that molybdenum alloying did not completely prevent the initiation of dynamic recrystallization. For analyzing the critical strain triggering dynamic recrystallization, single pass deformation cycles were interrupted at lower strain values. Dynamically recrystallized grains were not found for a strain of *ε* = 1 (Figure 9a,b). At the strain of peak stress, *ε*_p_ (being 1.26 and 1.30 for the CMnNbB and CMnNbMoB steels, respectively), dynamic recrystallization did occur (Figure 9c,d). The critical strain, *ε*c, triggering dynamic recrystallization was determined to be 1.08 and 1.1 for the CMnNbB and CMnNbMoB steels, respectively. The ratio of critical strain to peak strain was around 0.85 for both steels; this ratio is in the range of those reported in the literature for C–Mn and microalloyed steels [12]. Thus, the critical strain was obviously not significantly influenced by molybdenum alloying. However, molybdenum appeared to strongly obstruct the nucleation and growth of austenite grains under dynamic recrystallization conditions.

## 4. Discussion

The kinetics of dynamic recrystallization is influenced by the initial austenite grain size and the Zener–Hollomon parameter defined as Z = $\dot{\varepsilon }$exp(Q_def_/RT), where Q_def_ is the activation energy, R is the gas constant, $\dot{\varepsilon }$ is the strain rate, and T is the absolute temperature [13]. For coarse grain sizes, DRX kinetics is delayed due to the reduction of the amount of available nucleation sites as the grain boundary area per unit volume is decreased. No significant difference regarding the initial average austenite grain size after roughing simulation was observed in the studied steels. The measured values were approximately 50 µm in all steels. Therefore, the main influencing factor in the kinetics of DRX in the present experiments must have been the Zener–Hollomon parameter. Since the strain rate and deformation temperature were equal in all experiments, the activation energy was the main criterion accounting for the observed differences. The solutes having variable influence on the activation energy were Nb and Mo. Most of the Ti was bound in TiN, and a residual fraction participated in strain-induced NbC particles, so the residual solute amount of Ti was negligibly small. Solute Mo is known to impede the movement of high angle grain boundaries due to solute drag [14]. Schambron et al. [15] experimentally demonstrated that the activation energy for DRX correlates with Mo content (in mass percent) by a factor of 323 kJ/mol. Hence, the studied Mo content of 0.5 mass% increased the activation energy by 160 kJ/mol. Niobium is considered the most potent element in retarding DRX by solute drag [13]. However, under the applied hot deformation conditions, Nb partially precipitated and thereby lowered its solute content. From previous experiments on the same steels, it was concluded that the amount of soluble Nb after quenching hot worked steel is below 0.01 mass%. Strain-induced precipitates, on the other hand, are not very effective in suppressing DRX. Accordingly, DRX is expected to more likely occur in the CMnNbB steel, in agreement with the present experimental observations. Boron has been claimed to facilitate a softening effect due to its non-equilibrium grain boundary segregation [16,17]. A similar effect must have occurred in all investigated steels because the boron content was nominally identical.

**Figure 10:**
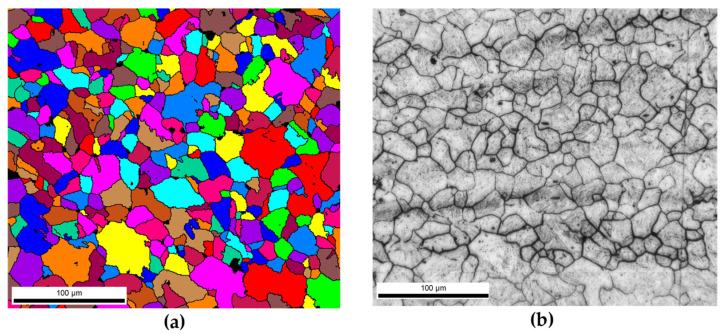
(**a**) Reconstructed austenite by EBSD and (**b**) optical image corresponding to austenite obtained after picric acid for CMnB steel grade.

The T_nr_ temperatures for the current steels were previously determined as 955, 980, 1010, and 1024 °C for the CMnB, CMnNbB, CMnMoB, and CMnNbMoB steels, respectively [4]. Accordingly, all finishing temperatures shown in the current experiments were below T_nr_. Though the Nb- and/or Mo-alloyed steels presented a pronouncedly pancaked austenite structure (Figure 5), the CMnB base steel comprised a completely equiaxed austenite microstructure with a generally fine average grain size (Figure 10). Due to this refinement, the total austenite grain boundary area per material volume was significantly increased. It was shown by de Rosa et al. [18] that boron segregation to austenite grain boundaries occurs extremely quickly and even during the quenching cycle. Several studies using atom probe tomography [19,20] have indicated a high concentration of boron in the immediate vicinity of austenite grain boundaries while the grain interior away from the boundaries becomes nearly depleted of boron. When boron segregation proceeds during austenite conditioning, additionally supported by a strong flux of vacancies towards the austenite boundaries, it is possible that no diffusible boron is left for covering the new austenite grain boundaries generated by dynamic recrystallization. Earlier investigations on the current and other boron-alloyed steels [2,6,7,8] revealed that the hardenability effect related to boron is weakened when applying substantial strain immediately before quenching. In CCT diagrams reported in the previous works, the ferrite and bainite phase fields are then shifted towards shorter times and higher transformation temperatures under direct quenching conditions.

In the CMnNbB steel, DRX fully occurred only when large strain (*ε* = 4) was applied. Thus, under conditions representing a TMCP rolling schedule, pancaked grains with accumulated strain could coexist with fine-sized recrystallized grains. The strain accumulation increased the driving force for transformation while the recrystallized grain fraction was exposed to insufficient boron protection, as described before. The presence of ferrite colonies (Figure 3) in the former partially recrystallized austenite area indicated an early transformation, probably due to insufficient boron protection. The combination of both effects further extended the ferrite phase field under direct quenching conditions compared to completely recrystallized CMnB steel [6].

The molybdenum-alloyed steels showed high strain accumulation without causing obvious dynamic recrystallization for the simulated rolling schedule (Figure 4). Their hardenability is much better than that of the Mo-free variants [6]. Ferrite formation was substantially retarded to a similar extent for direct quenching and conventional quenching conditions. That means the ferrite-suppressing effect of molybdenum was not measurably influenced by the presence of accumulated strain. This behavior is in good agreement with the results of Hannula et al. [7] who investigated the hardenability of very similar DQ steel alloys. Their results indicated the formation very fine-grained equiaxed austenite decorating the boundaries of much larger pancaked austenite grains when finish rolling at 800 °C. Such necklace structures gradually disappeared when molybdenum was added to the CMnB steel in amounts of 0.25 and 0.5 mass%. They were generally absent when applying less severe austenite pancaking and using a higher finish rolling temperature of 900 °C. However, despite severe pancaking and strain accumulation at the low finishing temperature, full hardenability was achieved in the molybdenum-alloyed steels in contrast to the CMnB steel. Like boron, molybdenum is strongly segregating at austenite grain boundaries [19,20]. Yet, a sufficiently high concentration level remained in the grain interior due to the comparably high molybdenum alloy addition. Accordingly, the Zener–Hollomon parameter had to have been significantly increased in the region near the grain boundary.

The micrographs of Figure 4 indicate that the austenite microstructure was not homogeneously deformed after the simulated rolling schedule but comprised a considerable range of austenite pancake thicknesses. Dynamic recrystallization was found to primarily initiate in the zones of most narrow pancakes. This was similar in all investigated steels (Figure 8a–c). The stress–strain curve for large deformation (Figure 7d) reveals that the stress initially increased to a relatively broad peak stress and then slowly declined to a steady state at large strain. In the steady state, the recrystallized grains generally grew in the CMnNbB steel, while they remained much finer in the molybdenum-alloyed steels (Figure 8 d–f). The observed features suggest geometric dynamic recrystallization (GDRX) as the acting mechanism [21]. In this mechanism, the impingement of serrated austenite pancake boundaries occurred when the pancake thickness approached 1–2 sub-grain size dimensions. Furthermore, the micrographs in Figure 9 indicate that GDRX did not instantaneously occur upon reaching the critical stress. The narrow ends of pancakes show the first appearance of ultrafine equiaxed grains in agreement with the model of de Pari and Misiolek [22], suggesting the gradual progress of GDRX with increasing strain. The steady state sub-grain size decreased with the increasing Zener–Hollomon parameter in the molybdenum-alloyed steels compared to the CMnNbB steel. In areas where extremely thin pancakes were clustered prior to GDRX, the molybdenum concentration must have been increased due to the close proximity of boundary segregation profiles. Therefore, the recrystallized grain size remained smaller in these areas, while it was larger in areas of formerly thicker pancakes. Petterson et al. [23], using hot torsion tests, showed that the critical strain for GDRX is directly related to the initial grain size and inversely related to the sub-grain size. Both features were similar in the currently discussed steels, as were the observed critical strains regardless of the Zener–Hollomon parameter. The critical strain triggering GDRX under deformation in compression was indicated to become smaller than that in torsion [23], which is of relevance when considering industrial rolling.

The occurrence of partial GDRX before quenching has additional implications for martensitic transformation behavior. Experimental and theoretical studies [24,25,26] have indicated that the martensite-start temperature rapidly decreases for prior austenite grain sizes below 10 µm. Equiaxed grains originating from GDRX have sizes in the order of 1 µm and hence transform at lower temperature than surrounding austenite pancakes. The delayed transformation of the ultrafine austenite grains during quenching can thus lead to the buildup of residual stress because the dilatation caused by the martensitic transformation cannot be accommodated by plastic deformation in the previously transformed martensitic matrix. Such residual stresses typically result in unwanted quench distortion [27] and can have a negative impact regarding hydrogen-induced delayed cracking [28].

## 5. Conclusions

The current study has confirmed that dynamic recrystallization (DRX) can occur in direct quenching steels during austenite conditioning at low temperatures. After applying a typical TMCP deformation-temperature schedule to standard CMnB steel, DRX resulted in a fully equiaxed microstructure. The microalloying of niobium (0.026%) to such steel produced pancaked austenite with the localized appearance of very fine equiaxed austenite grains originating from DRX. The addition of molybdenum (0.5%) completely suppressed DRX under the same TMCP conditions. This effect of molybdenum was related to a significant increase of the Zener–Hollomon parameter.

Specific austenite conditioning, applying larger strain at low austenite temperature, demonstrated that the molybdenum-alloyed steel could experience DRX, resulting in ultrafine austenite grains. The progress of DRX and the austenite grain size was evidently smaller than in the niobium-microalloyed steel without molybdenum addition.

The initiation of DRX in the niobium- and molybdenum-added steels was related to the mechanism of geometrical DRX (GDRX). GDRX appeared to occur when the thickness of individual austenite pancakes was approaching the dimension of 1–2 sub-grains and proceeded gradually. Under a large single-pass strain (*ε* = 4), DRX was completed in the Nb-microalloyed steel but not in the molybdenum-alloyed steels. The larger Zener–Hollomon parameter in the latter steels resulted in a smaller size of the recrystallized grains.

The presence of fine-grained austenite generated by DRX was shown to produce soft phases upon quenching under an industrial cooling rate of 30 °C/s. It was argued that the sudden and late increase of the austenite grain boundary area caused by DRX could weaken the hardenability effect related to boron.

Molybdenum alloying acts twofold—by its high inherent hardenability effect and by avoiding DRX.

The presence of fine-grained austenite originating from DRX within a partially coarser microstructure is expected to cause non-synchronous martensite transformation, with the fine grains transforming at lower temperature. This phenomenon can induce residual stresses that lead to quench distortion and should be investigated in a dedicated study.

## Figures and Tables

**Table 1:** Chemical composition of the steels investigated in this work (in weight percent).

Steel	C	Si	Mn	Mo	Nb	B
CMnB	0.15	0.32	1.05	-	-	0.0022
CMnNbB	0.16	0.29	1.05	-	0.026	0.0019
CMnMoB	0.16	0.28	1.07	0.5	-	0.0022
CMnNbMoB	0.16	0.31	1.07	0.5	0..026	0.0018

## Data Availability

The data presented in this study are available on request from the corresponding author. The data are not publicly available due to project confidentiality.
